# Dengue Envelope Protein as a Cytotoxic Factor Inducing Hemorrhage and Endothelial Cell Death in Mice

**DOI:** 10.3390/ijms251910858

**Published:** 2024-10-09

**Authors:** Te-Sheng Lien, Der-Shan Sun, Wen-Sheng Wu, Hsin-Hou Chang

**Affiliations:** 1 Department of Molecular Biology and Human Genetics, Tzu-Chi University, Hualien 970, Taiwan; alan211@mail.tcu.edu.tw (T.-S.L.); dssun@mail.tcu.edu.tw (D.-S.S.); 2 Division of General Surgery, Department of Surgery, Hualien Tzu Chi Hospital, Buddhist Tzu Chi Medical Foundation, Hualien 970, Taiwan; wuws@gms.tcu.edu.tw; 3 Department of Laboratory Medicine and Biotechnology, College of Medicine, Tzu Chi University, Hualien 970, Taiwan

**Keywords:** endothelial cell apoptosis, caspase-3, dengue hemorrhagic fever, envelope protein domain III, z-DEVD-FMK, chondroitin sulfate B, N-acetyl cysteine

## Abstract

Dengue virus (DENV) infection, prevalent in tropical and subtropical regions, can progress to dengue hemorrhagic fever (DHF), which increases mortality during secondary infections. DHF is characterized by endothelial damage and vascular leakage. Despite its severity, no specific antiviral treatments exist, and the viral factors responsible for endothelial damage remain unclear. This study examines the role of the DENV envelope protein domain III (EIII) in inducing endothelial apoptosis using a mouse model. Additionally, we aim to explore whether cell death-inducing pathways could serve as drug targets to ameliorate EIII-induced endothelial injury and hemorrhage. In vitro experiments using human endothelial HMEC-1 cells demonstrated that both recombinant EIII (rEIII) and DENV markedly induced caspase-3-mediated endothelial cell death, an effect that was attenuated by co-treatment with chondroitin sulfate B (CSB), N-acetyl cysteine (NAC), and the caspase-3 inhibitor z-DEVD-FMK. In vivo, sequential injections of rEIII and anti-platelet immunoglobulin in mice, designed to mimic the clinical phase of DHF with peak viremia followed by an increase in DENV-induced Ig, including autoantibodies, revealed that these dual treatments markedly triggered caspase-3-dependent apoptosis in vascular endothelial cells at hemorrhage sites. Treatments with z-DEVD-FMK effectively reduced DHF-like symptoms such as thrombocytopenia, hemorrhage, inflammation, hypercoagulation, and endothelial damage. Additionally, CSB and NAC alleviated hemorrhagic symptoms in the mice. These results suggest that targeting EIII, reactive oxygen species, and caspase-3-mediated apoptosis could offer potential therapeutic strategies for addressing EIII-induced hemorrhagic pathogenesis.

## 1. Introduction

Dengue virus (DENV) infection is one of the most rapidly growing and significant mosquito-borne infections in tropical and subtropical regions worldwide [1,2]. While primary DENV infection is typically self-limiting and may cause flu-like symptoms, secondary infections are associated with a higher risk of severe dengue, also known as dengue hemorrhagic fever (DHF). Currently, there are no specific antiviral treatments for DENV infection. Climate change, leading to variations in temperature and humidity, is likely expanding mosquito habitats and increasing the overall risk and mortality of DENV infections [3,4,5,6,7,8,9,10].

Severe dengue is characterized by endothelial damage and vascular leakage [11,12,13,14,15,16,17,18,19,20,21,22], which usually occur 3–6 days after the onset of disease during the acute phase of DHF [22,23]. The occurrence of this phase after the period of decreasing viremia suggests that the severe symptoms are due to endothelial injury rather than direct infection and replication of the DENV in the endothelium [22]. However, the specific viral factors causing vascular endothelial damage remain unclear.

The DENV envelope protein (E) domain III (EIII) is known for its role in host cell surface protein binding during viral entry [24]. Our previous in vitro studies showed that treatment with viral-load-equivalent levels of recombinant EIII (rEIII) can induce various regulated cell death (RCD) responses in endothelial cells, platelets, and neutrophils, indicating potential cytotoxicity of rEIII when it binds to these cells [25,26,27].

Given that secondary, but not primary, DENV infections are associated with DHF, and that abnormal immune responses are more intensely elicited during secondary infections, it is suggested that DHF is characterized not only by direct viral damage but also by these critical pathogenic immune responses [28,29,30,31,32,33]. DENV infection and immunization with viral protein nonstructural protein (NS1) can lead to the production of autoantibodies [e.g., anti-platelet immunoglobulins (Igs)], contributing to abnormal immune responses and excessive inflammation [29,31,34,35,36,37,38]. As vascular leakage follows peak viremia (EIII expressed on virion surfaces) and coincides with a marked increase in DENV-elicited antibodies [39], it is plausible that the sequential elevation of circulating EIII levels combined with DENV-elicited antibodies through injections results in endothelial damage and hemorrhage pathogenesis in vivo.

In previous studies, we demonstrated that sequential injections of DENV, rEIII, and anti-platelet Ig, mimicking the clinical phase of DHF with peak viremia followed by elevated DENV-induced Ig and autoantibodies [25,33,35], could induce hemorrhagic pathogenesis in mice. Intriguingly, this model replicates key DHF-associated responses, including vascular leakage, hemorrhage, thrombocytopenia, and excessive inflammation [25,33,35]. In this scenario, however, the specific form of RCD, such as apoptosis, contributing to endothelial cell death in vivo has not been clearly established. Therefore, our study aims to determine the role of apoptosis and explore whether it could be targeted for treating the combined effects of rEIII and autoantibodies in mice.

Experiments have used an increased expression of FcγRIII and tissue factor (TF), along with reduced thrombomodulin (TM) levels, as indicators of endothelial inflammation and damage [40,41,42,43], while active caspase-3 serves as a marker of endothelial apoptotic cell death [44,45]. In this study, to assess the cytotoxic effects of EIII on endothelial cells, we measured the expression levels of FcγRIII, TF, TM, and activated caspase-3 in cultured endothelial cells treated with rEIII and DENV virions. In the in vivo experiments, we examined whether sequential injections of rEIII and anti-platelet Ig in a mouse model could induce endothelial apoptosis associated with hemorrhage.

Our aim is to explore whether targeting endothelial cell death-inducing pathways could serve as a therapeutic approach to ameliorate EIII-induced endothelial injury and hemorrhage. To test this, we used inhibitors such as chondroitin sulfate B (CSB), which disrupts DENV-EIII binding [26,27], N-acetyl cysteine (NAC), an antioxidant that reduces oxidative stress during DENV infection [46], and a caspase-3 inhibitor z-DEVD that blocks apoptotic cell death [47,48,49,50]. This approach was based on our preliminary findings that rEIII induces endothelial apoptosis in vitro and that the combination of rEIII and autoantibodies causes endothelial apoptosis in mice, prompting the inclusion of a caspase-3 inhibitor in the study. Additionally, our previous research demonstrated that CSB, which blocks rEIII-endothelial interactions [27], and NAC, an antioxidant, suppressed rEIII-induced endothelial pyroptosis [25]. However, their ability to prevent rEIII-induced endothelial apoptosis was not yet clear. Thus, we used these agents to explore their effects on rEIII- and rEIII plus autoantibody-induced endothelial abnormalities in both the in vitro and in vivo models, with promising results in reducing endothelial damage. The regulatory mechanisms and therapeutic potential are discussed further.

## 2. Results

### 2.1. Treatments of Recombinant EIII Protein and Virion of DENV Markedly Induced Elevation of Endothelial Inflammation and Damage Markers in HMEC-1 Endothelial Cells

**Figure 1:**
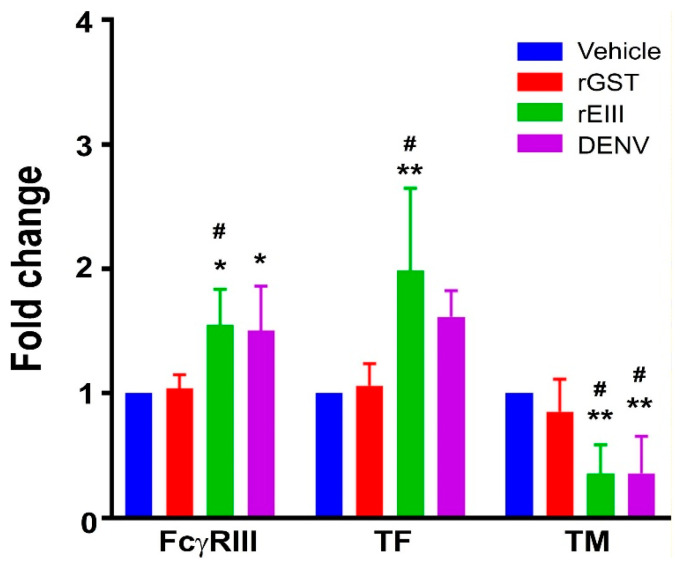
Assessment of endothelial cell inflammation and damage markers. Cell populations with relatively high surface expression levels of endothelial cell inflammation and damage markers, FcγRIII, tissue factor (TF), and thrombomodulin (TM), of HMEC-1 cells were measured using flow cytometry following treatments with vehicle (solvent control), recombinant glutathione S-transferase (rGST), rEIII, and DENV. Vehicle control groups were set to a 1-fold baseline for normalization. Statistical significance is indicated by ** for *p* < 0.01 and * for *p* < 0.05, compared to untreated vehicle groups, and # for *p* < 0.05, compared to rGST groups. This study consisted of three experiments with two replicates per group (*n* = 6).

Employing a reductionist approach, we compared the effects of the rEIII and DENV virion treatments on human endothelial HMEC-1 cells to determine if rEIII is a key viral cytotoxic factor in DENV-induced endothelial pathogenesis. We assessed the surface expression of the endothelial markers FcγRIII, TF, and TM, which indicate inflammation and damage. Both rEIII and DENV virion treatments resulted in increased FcγRIII and TF expression, along with decreased TM levels (Figure 1; examples of flow cytometry gating for TM expression can be found in Figure S1). This aligns with previous studies showing that elevated FcγRIII and TF expression and reduced TM serve as indicators of endothelial inflammation and damage, respectively [40,41,42,43]. These results suggest that rEIII, a DENV-associated cytotoxic factor, can effectively induce endothelial inflammation and damage.

### 2.2. Treatments of Recombinant EIII Protein and Virion of DENV Markedly Induced Caspase-3-Associated Cell Death in HMEC-1 Endothelial Cells

**Figure 2:**
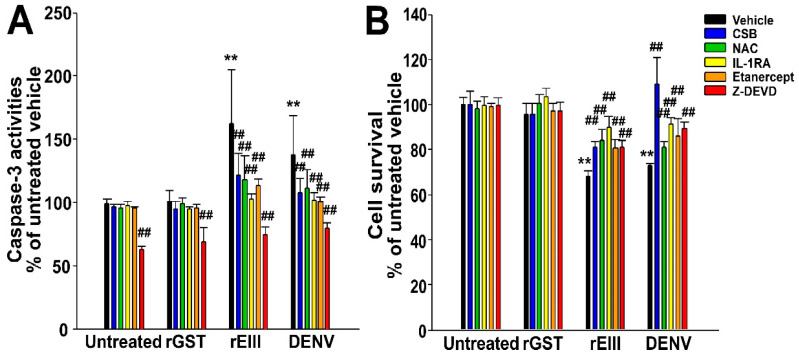
Caspase-3-associated cell death occurred after HMEC-1 human microvascular endothelial cells were challenged with recombinant EIII (rEIII) and DENV virions. The recombinant rEIII and DENV treatments were compared to untreated groups and control groups treated with the recombinant protein rGST. The rescue effects of chondroitin sulfate B (CSB), antioxidant N-acetyl cysteine (NAC), IL-1 receptor antagonist (IL-1RA), TNF-α inhibitor etanercept, and caspase-3 inhibitor z-DEVD-FMK (z-DEVD) were assessed by measuring the suppression of cellular caspase-3 activities (**A**) and the reduction in cell death (**B**), compared to untreated groups. The untreated vehicle groups were set to 100% for normalization. ** indicates a statistically significant exacerbated effect at *p* < 0.01 compared to untreated vehicle groups; ## indicates a statistically significant ameliorated effect at *p* < 0.01 compared to respective vehicle groups. This study involved three experiments with two replicates per group (*n* = 6).

We aimed to evaluate whether inhibitors of cell death pathways could serve as targeted therapeutic agents. However, the expression levels of FcγRIII, TF, and TM do not provide insights into cell death conditions (Figure 1). Consequently, we measured caspase-3 activity and cell survival in HMEC-1 cells following treatments with rEIII and DENV virus particles (Figure 2). We found that additional treatments with CSB, antioxidant NAC, interleukin (IL)-1 receptor antagonist (IL-1RA), tumor necrosis factor-α (TNF-α) inhibitor etanercept, and caspase-3 inhibitor z-DEVD-FMK (z-DEVD) markedly rescued cells from rEIII-, and DENV-induced caspase-3-associated death (Figure 2).

### 2.3. Treatment with Recombinant EIII Protein and Anti-Platelet Ig in a Mouse Model Markedly Induced Caspase-3-Associated Damage in the Vascular Endothelial Cells of Hemorrhage Lesions

**Figure 3:**
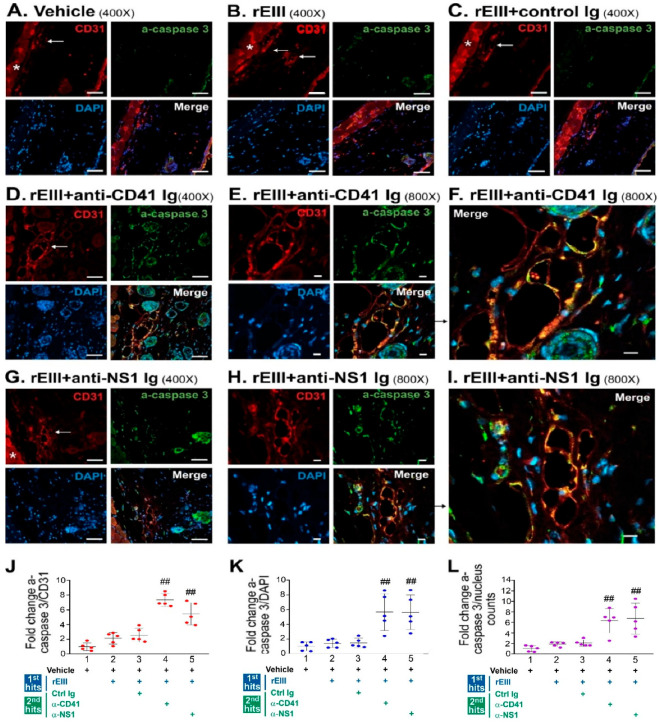
Immunohistochemistry analysis of endothelial damage in hemorrhage lesions of mouse skin samples using confocal microscopy. Mouse skin samples were collected after subcutaneously challenged with vehicle (**A**), rEIII (first hit alone) (**B**), rEIII + control Ig (**C**), rEIII + anti-CD41 Ig (dual challenge) (**D**–**F**), and rEIII + anti-NS1 Ig (dual challenge) (**G**–**I**). Skin samples were then subjected to immunohistochemistry (IHC) (**A**–**I**) and quantitative (**J**–**L**) analyses. CD31, activated caspase-3 (a-caspase-3), and DAPI staining signals in the IHC images indicate the localizations of endothelial cells, apoptotic cells, and cell nuclei in the mouse skin sections, respectively. The asterisks (*) in (**A**–**C**,**G**) (red channels) mark fluorescence signals from muscle layers underneath the skin. White arrows in (**A**–**D**,**G**) (CD31 staining) indicate the locations of blood vessels. Panels (**F**) and (**I**) are enlarged merged images shown in (**E**) and (**H**), respectively. Scale bars: (**A**–**D**,**G**) 50 μm; (**E**,**F**,**H**,**I**) 10 μm. Quantitative results were obtained by Image J-abstracted signal intensities from respective channel images: activated caspase-3 (green channel)/CD31 (red channel) (**J**), activated caspase-3 (green channel)/DAPI (blue channel) (**K**), and activated caspase-3 (green channel)/nucleus counts (accountable nucleus number in the blue channel) (**L**), from five different 200× images (*n* = 5, in 2 independent experiments). Due to the high fluorescence background, muscle areas were excluded from quantitative analyses. Vehicle groups were normalized to 1-fold (**J**–**L**). ## *p* < 0.01 vs. vehicle groups.

Although our experiments with rEIII and anti-platelet Ig treatments have demonstrated that these dual challenges could induce hemorrhagic pathogenesis in mice [25], it remains unclear whether these treatments induce apoptosis in vivo. Given that the rEIII and DENV virions elicited similar pathogenic responses in endothelial cells (Figure 1 and Figure 2), we used a reductionist approach to evaluate if rEIII can induce hemorrhage-associated apoptotic cell death in vascular endothelial cells in vivo. In our mouse model, we applied dual challenges with rEIII and anti-platelet Ig and compared the outcomes with those from previously reported methods involving DENV virion and anti-platelet Ig dual challenges [35]. We employed immunohistochemistry (IHC) fluorescent staining to detect the endothelial cell marker CD31 and activated caspase-3 at hemorrhage lesion sites (Figure 3).

We found that rEIII plus anti-platelet Igs (either anti-CD41 monoclonal Ig MWReg30 [51], or anti-DENV-nonstructural protein 1 Ig (anti-NS1 Ig), which contains anti-platelet Ig fractions [31,35]) induced a clear appearance and co-localization of CD31 and activated caspase-3 signals in the IHC fluorescent staining (Figure 3A–I, rEIII + control Ig groups vs. rEIII + anti-CD41 Ig, and rEIII + anti-NS1 Ig groups), with markedly elevated quantitative results (Figure 3J–L). These results suggest that rEIII plus the anti-platelet Ig challenges effectively induce caspase-3-associated apoptotic cell death in endothelial cells in vivo.

### 2.4. Treatment with Caspase-3 Inhibitor z-DEVD Markedly Mitigated Hemorrhage-Associated Pathogenesis Induced by the Recombinant EIII Protein and Anti-Platelet Ig Challenges in Mice

**Figure 4:**
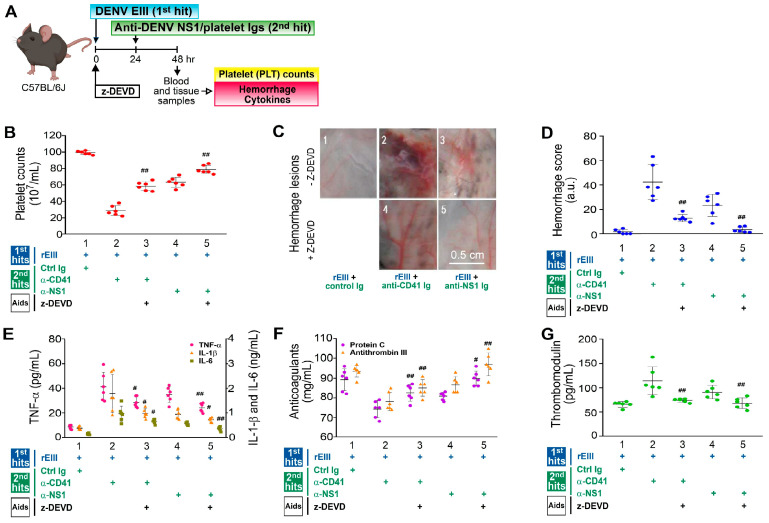
Involvement in the caspase-3 pathway in pathogenesis induced by rEIII and autoantibody treatments is illustrated. The experimental outline (**A**) and various parameters, including mouse platelet counts (**B**), hemorrhagic lesion severity (**C**,**D**), proinflammatory cytokines TNF-α, IL-1β, and IL-6 (**E**), anticoagulants protein C and antithrombin III (**F**), and soluble thrombomodulin (**G**), are shown. Treatment with the caspase-3 inhibitor z-DEVD (**B**–**G**) reduced hemorrhage and inflammation in wild-type mice challenged with rEIII (the first hit) and autoantibodies [the second hit: anti-CD41 Ig (α-CD41) and anti-NS1 Ig (α-NS1), containing anti-platelet fractions]. This study used six mice per group, with three experiments conducted (*n* = 6). Data are presented as mean ± SE, with # *p* < 0.05 and ## *p* < 0.01 indicating significant rescue compared to the respective rEIII + α-CD41/α-NS1 groups. Ctrl Ig: control Ig. For detailed hemorrhage scoring, refer to Figure S2. The mouse diagram was created using Biorender.com.

The caspase-3 inhibitor z-DEVD was used to determine whether caspase-3-dependent apoptosis contributed to the hemorrhage pathogenesis induced in vivo by the rEIII and autoantibody challenges, in line with our previously established mouse model [25]. While applying human guidelines to assess disease severity in mice is challenging, the pathophysiological alterations observed in this model bear some resemblance to those seen in DHF patients [35]. Following the dual challenges with rEIII plus either an anti-platelet monoclonal antibody or anti-dengue nonstructural protein 1 immunoglobulin (anti-NS1 Ig, which includes anti-platelet autoantibody fractions), the z-DEVD treatment markedly ameliorated all measured pathological manifestations in the mice (Figure 4). These included thrombocytopenia (Figure 4A outline, Figure 4B), hemorrhage (Figure 4C,D), inflammation (Figure 4E), hypercoagulation (Figure 4F), and endothelial damage (Figure 4G)—symptoms linked to DHF [52,53,54]. This implies that the caspase-3 pathway is involved in the disease mechanism, with caspase-3-associated cell death contributing to the pathogenesis driven by the rEIII and autoantibody challenges.

### 2.5. Treatment with CSB and NAC Markedly Rescued Hemorrhage-Associated Pathogenesis Induced by Recombinant EIII Protein and Anti-Platelet Ig Treatments in Mice

**Figure 5:**
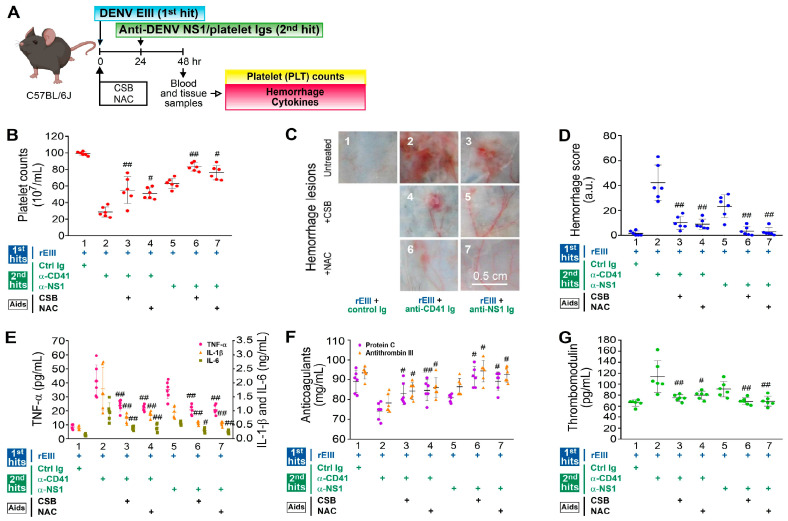
Rescue of pathogenesis induced by rEIII plus autoantibody dual challenges by CSB and NAC. The experimental layout (**A**) and the pathophysiological alterations in mouse platelet counts (**B**), hemorrhagic lesions (**C**,**D**), proinflammatory cytokines TNF-α, IL-1β, and IL-6 (**E**), anticoagulants protein C and antithrombin III (**F**), and soluble thrombomodulin (**G**) are depicted. (**B**–**G**) Treatments with chondroitin sulfate B (CSB), a glycosaminoglycan that binds to rEIII (0.5 mg/kg), and N-acetyl cysteine (NAC), a scavenger of reactive oxygen species (ROS) (50 mg/kg), were employed to alleviate hemorrhage and inflammation in wild-type (WT) mice subjected to the “rEIII (the first hit) plus autoantibody (the second hit)” dual challenge (panels 3 and 4, 6 and 7 vs. 2 and 5, respectively). The data are presented as mean ± SE. # *p* < 0.05 and ## *p* < 0.01 indicate significant rescue compared to the respective “rEIII + anti-CD41 Ig” or “rEIII + NS1 Ig” groups. Ctrl Ig: control Ig. This study included six mice per group, with a total of three experiments conducted (*n* = 6). The mouse diagram was created using Biorender.com.

As the treatment with CSB and NAC appeared to potentially rescue caspase-3-associated endothelial cell death in vitro (Figure 2), we aim to explore whether CSB and NAC treatments can also mitigate hemorrhage pathogenesis in vivo. Our findings revealed that treatments with CSB and NAC markedly ameliorated various pathological changes, including thrombocytopenia (outlined in Figure 5A, shown in Figure 5B), hemorrhage (Figure 5C,D), inflammation (Figure 5E), hypercoagulation (Figure 5F), and endothelial damage (Figure 5G), which are potential clinical indicators associated with DHF [52,53,54], induced by the dual challenge protocol.

In summary, our injection protocol in the mouse model, where rEIII is administered and initially followed by anti-platelet Ig 24 h later, somewhat reflects the clinical progression of DHF, in which viremia precedes the induction of DENV-associated autoantibodies and hemorrhage pathogenesis develops when both viremia and autoantibodies are present. Given that EIII-induced caspase-3 endothelial apoptosis is likely implicated in the endothelial cell damage (Figure 2), treatments with inhibitors like CSB, which target EIII-cell-surface binding, as well as the ROS scavenger NAC, and the caspase-3 inhibitor z-DEVD, which targets endothelial cell death (Figure 2, Figure 4 and Figure 5), may offer promising avenues for developing therapeutic strategies to mitigate hemorrhage pathogenesis induced by the dual challenges.

## 3. Discussion

In this study, we have demonstrated that recombinant DENV EIII, in conjunction with the autoantibody dual challenges, can induce caspase-3-dependent apoptosis in endothelial cells both in vitro and in vivo. This finding provides new insights into the molecular interactions between the DENV and host cell responses, potentially elucidating the mechanisms behind the vascular complications observed in severe DENV infections.

Dengue viruses typically do not induce DHF in wild-type mice through natural propagation [55,56]. However, following the established protocols, we can induce hemorrhagic pathogenesis by concurrently administering DHF-viral-load DENV and anti-platelet autoantibodies, leading to severe hemorrhage and a surge in cytokines [25,35]. In this study, we applied a reductionist approach and found that treatment with rEIII alone effectively substitutes DENV in inducing endothelial damage, as demonstrated in both the in vitro endothelial cell culture model (Figure 1 and Figure 2) and the dual challenge mouse model (Figure 3, Figure 4 and Figure 5), in comparison to DENV plus the autoantibody treatments from a previous report [35].

To better mimic the pathological changes in mice that resemble human responses, we employed a DHF-viral-load equivalent EIII dosage, following previously described methods [27]. This approach involved comparing the functionally equivalent levels of DENV and rEIII using an activated partial thromboplastin time (aPTT) plasma clotting assay, as rEIII can inhibit the anticoagulant effect of heparin [27]. To explore the dose-dependent response of DENV and rEIII on plasma clotting in heparinized mouse plasma, regression curves were plotted, comparing the DENV and rEIII dosages with the aPTT results. We found that both the DENV and rEIII doses were inversely correlated with the aPTT in a similar manner. Through this method, we determined the functionally equivalent doses of DENV and rEIII in their interaction with heparin. For instance, the estimated DHF viral load virion titer (1 × 10^3^ to 1 × 10⁶ PFU) [57] roughly corresponds to 1.7 × 10^−3^ to 1.2 μM rEIII. The 0.3–1.2 μM rEIII dose used for the analysis in this study falls within this range.

Our findings revealed that rEIII alone is sufficient to induce comparable levels of caspase-3 activation and apoptotic cell death in vitro, similar to the DENV virion treatments, indicating that virus replication is not required to cause endothelial cell damage. Notably, our in vivo protocol—administering rEIII followed by anti-platelet Ig 24 h later—in part mirrors the clinical progression of DHF, where viremia precedes DENV antibody production [39]. This sequence effectively induces caspase-3-associated endothelial cell damage (Figure 3) and hemorrhagic pathogenesis in mice (Figure 4 and Figure 5), resembling the traditional LPS-elicited Shwartzman reaction [35,58,59,60], which triggers TNF-α, IL-1, pro-coagulation activity, and hemorrhaging (Figure 4 and Figure 5). These results suggest that dengue-associated hemorrhage may stem from an rEIII and autoantibody-induced Shwartzman reaction-like response. Notably, compared to monoclonal anti-CD41 Ig, anti-NS1 Ig exhibited relatively lower potency in hemorrhage-related pathogenesis, such as the hemorrhage score, proinflammatory cytokine induction, and thrombomodulin release (Figure 4 and Figure 5). This may be attributed to the fact that, unlike monoclonal anti-CD41 Ig, only specific fractions of anti-NS1 Ig demonstrate anti-platelet activity, resulting in a less pronounced pathogenic effect in mice.

Endothelial dysfunction and vascular leakage are key characteristics of severe dengue disease, including DHF and dengue shock syndrome [23,61]. Previous studies have shown that elevated FcγRIII and TF expression, along with reduced TM levels, are markers of endothelial inflammation and damage [40,41,42,43]. Consistent with these observations, our findings demonstrate that the rEIII and DENV virion treatments lead to an increased expression of FcγRIII and TF and decreased TM expression, which are linked to endothelial apoptosis in vitro.

Endothelial apoptosis induced by DENV and DENV-elicited autoantibodies has been reported in various in vitro models [46,62,63,64,65,66,67,68], while the specific form of RCD, such as apoptosis, contributing to endothelial cell death in vivo remains unclear. In this study, we demonstrate for the first time that rEIII induces endothelial apoptosis in an rEIII plus autoantibody dual challenge mouse model. The marked rescue effect of the caspase-3 inhibitor z-DEVD, which prevents caspase-3 activation, suggests that apoptosis plays a critical role in initiating hemorrhagic pathogenesis. Despite DENV’s low replication levels in endothelial cells, indicating that endothelial damage is not solely due to viral replication, our findings show that rEIII alone can induce endothelial cell death, further supporting the idea that DENV replication is unnecessary for this damage. The caspase-3-mediated cell death observed in the cultured HMEC-1 cells, which was mitigated by the caspase-3 inhibitors, indicates that rEIII triggers the apoptotic pathway. Additionally, rEIII plus the autoantibody dual challenges induced apoptotic abnormalities in the CD31^+^ vascular endothelial cells at hemorrhagic lesions in the mouse model. Previous studies have demonstrated that CSB can inhibit EIII binding to host cells [25], which explains the rescue effects of CSB observed in both the in vitro and in vivo experiments (Figure 2 and Figure 5).

In this present study, we found that NAC, IL-1RA, and etanercept, which suppress ROS and the proinflammatory cytokines IL-1 and TNF-α, respectively, were able to rescue rEIII-induced caspase-3-associated cell death (Figure 2). This suggests that rEIII-induced caspase-3 activation may be mediated through ROS and inflammation, leading to cellular disruption and apoptosis. While the beneficial effects of IL-1RA and etanercept were previously demonstrated [25], our current findings further highlight NAC as a potent agent in mitigating rEIII plus autoantibody-induced hemorrhagic pathogenesis in mice (Figure 5). Consistent with prior research, IL-1RA and etanercept enhance endothelial cell survival by blocking IL-1 and TNF-α, and NAC’s protective effect is plausible given the link between excessive ROS levels and apoptosis [69,70]. Additionally, the protective role of CSB, which blocks EIII’s interaction with target cells [26,27], is consistent with its ability to safeguard rEIII-treated endothelial cells. Further studies are needed to elucidate the precise molecular events following the rEIII–autoantibody interaction.

Given the role of rEIII and autoantibodies in inducing apoptosis, interventions that can block this interaction may serve as potential therapeutic strategies. Developing inhibitors to prevent rEIII and autoantibody-induced pathogenic effects could mitigate endothelial damage and improve patient outcomes. Our findings demonstrate that inhibiting rEIII binding with CSB, suppressing ROS with NAC, and blocking caspase-3 apoptosis with z-DEVD markedly rescue hemorrhagic pathogenesis in the mouse model. These results suggest that targeting EIII, ROS, and caspase-3 apoptosis may be promising approaches for developing drugs to treat DENV-induced hemorrhagic pathogenesis. Additionally, CSB, NAC, and z-DEVD could serve as promising reference compounds for the further development of therapeutic agents to control DHF.

Here, we demonstrate that the rEIII and autoantibody dual challenges successfully induce hemorrhagic pathogenesis in mice, which can be mitigated using various drugs and inhibitors. This model shows promise for studying the mechanisms of dual-challenge-induced pathogenesis and potential rescuing agents. However, since mice are not the native hosts of DENV and DENV cannot typically replicate in them, using cultured cells and mouse models may not fully capture the complex human immune response to DENV infection and DHF pathogenesis. Future research is needed to validate these findings in human subjects and explore the clinical relevance of EIII and autoantibodies in DHF pathogenesis.

## 4. Materials and Methods

### 4.1. DENV, Cell Lines, Recombinant Proteins, and Antibodies

The DENV (DENV2 strains PL046) and soluble recombinant proteins, such as DENV envelope protein domain III (rEIII) and nonstructural protein 1 (rNS1), were acquired and purified following previously established methods [31,71]. Mosquito C6/36 cells (ATCC-CRL-1660) were used for DENV amplification, and sucrose density gradient centrifugation was employed for DENV purification. Human microvascular endothelial cells (HMEC-1-CRL-3243) were used for the in vitro endothelial analyses. Quantification of the DENV copies and titers was performed using QIAamp viral RNA minikits (Qiagen, Hilden, Germany), Maxima SYBR Green qPCR Master Mixes (Thermo Scientific, Waltham, MA, USA), a Lightcycler 480 (Roche, Basel, Switzerland), and plaque-forming assays on the BHK-21 cells (ATCC-CCL-10) [72]. Preimmune Ig and anti-NS1 Ig from rabbits were obtained before and after the rNS1 immunizations, respectively [31,71]. Rabbit anti-NS1 sera were collected 7–10 days after the fifth immunization cycle. The IgG fractions were purified using a protein-A column connected to a peristaltic pump (Amersham Biosciences, Amersham, UK) operating at a flow rate of 0.5–1 mL min^−1^ from those rabbits with the highest anti-platelet IgG titers.

### 4.2. HMEC-1 Endothelial Cell Experiments

HMEC-1 cells (CRL-3243; obtained from the Centers for Disease Control and Prevention, Atlanta, GA, USA) were treated with either a vehicle control phosphate-buffered saline (PBS), rGST (1.6 µg/mL), rEIII (1.6 µg/mL), or DENV (1 × 10⁵ PFU/mL) at 37 °C for 24 h. Following treatment, the cells were detached using a non-enzymatic dissociation solution (Sigma-Aldrich, Burlington, MA, USA) to avoid trypsin digestion, which could alter the expression of the surface markers. The surface levels of FcγRIII, tissue factor (TF), and thrombomodulin (TM) on the suspended HMEC-1 cells were then assessed by flow cytometry. FcγRIII was labeled with phycoerythrin (PE)-conjugated anti-CD16 antibody (Biolegend, San Diego, CA, USA), while TF was marked using rabbit anti-TF Ig (Sekisui Diagnostica, Burlington, MA, USA), followed by fluorescein isothiocyanate (FITC)-conjugated goat anti-rabbit IgG (Jackson ImmunoResearch, West Grove, PA, USA). The TM was labeled using FITC-conjugated anti-human TM (Novus Biologicals, Centennial, CO, USA). After labeling for 1 h at 37 °C, the cells were washed with PBS and analyzed using a flow cytometer (FACSAria II, BD Biosciences, Franklin Lakes, NJ, USA).

To investigate the endothelial cell death induced by rEIII and DENV virion, the HMEC-1 cells were treated with the vehicle (normal saline), rGST (25 µg/mL), rEIII (25 µg/mL), or DENV (1 × 10⁵ PFU/mL) for 4.5 h, either alone or in combination with specific inhibitors. The cell-rescuing agents, including NAC (1 mM), IL-1RA (250 ng/mL), etanercept (100 µg/mL), and z-DEVD (10 µM), were pre-incubated with the cells for 0.5 h. A slight protocol variation was applied for CSB (10 µg/mL), which was co-incubated with rEIII, rGST, and DENV in PBS for 0.5 h before being added to the cells (Figure S3A). Following treatment, the HMEC-1 cells were washed with PBS to eliminate residual substances and were further incubated for an additional 72 h. After this incubation period, cell survival was assessed using a WST-1 kit (Roche Life Science, Penzberg, Germany), and cell apoptosis was determined using a caspase-3 activity kit (BioVision, Milpitas, CA, USA). For applying WST-1, 10 µL of the WST-1 reagent was added to each well of 96-well plates. The plates were incubated at 37 °C for an additional 30 min, allowing sufficient time for the conversion of WST-1 tetrazolium salt to formazan by metabolically active cells. After incubation, the absorbance of each well was measured at 450 nm using a microplate reader, with a reference wavelength of 620 nm (Varioskan Flash Multimode reader; Thermo Fisher Scientific, Waltham, MA, USA). The amount of formazan dye generated is directly proportional to the number of viable cells, and thus, the absorbance values were used to assess cell viability. Following the same treatment protocols as for the WST-1 assay, the cells were prepared for the caspase-3 activity assay. After treatments, the cells were harvested or lysed according to the assay protocol. A caspase-3-specific substrate was added to the cells. The mixture was incubated for 1 h at 37 °C, allowing caspase-3 to cleave the substrate, and then the fluorescence signals were determined using a microplate reader (Thermo Fisher Scientific). A summary of the experimental settings of the in vitro endothelial cell analyses is available in Figure S3.

### 4.3. Laboratory Mice

Male wild-type C57BL/6J mice, aged 8–12 weeks, were procured from the National Laboratory Animal Center in Taipei, Taiwan [73,74,75,76]. The mice were housed in the Animal Center of Tzu-Chi University, maintaining a specific pathogen-free environment with controlled lighting and temperature and provided with ad libitum access to filtered water and food. A total of 102 wild-type mice were used in the study. Ethical approval for all the experimental procedures involving the animals was obtained from the Animal Care and Use Committee of Tzu-Chi University in Hualien, Taiwan (approval ID: 110081).

### 4.4. Induction of a Local Shwartzman Reaction-like Response with Dual Challenges Using rEIII and Anti-Platelet Ig

To induce a local hemorrhage in the mice via the Shwartzman reaction, the conventional approach involves administering two consecutive subcutaneous injections of lipopolysaccharide (LPS) over two days [58]. In the dual challenge mouse model, rEIII (2 mg/kg, a dose equivalent to the viral load of the DHF virions containing surface EIII [25]) and anti-platelet Ig were injected sequentially at the same skin location, with a 24 h interval between injections. Both rEIII and antibodies were dissolved in PBS and injected into the mice at room temperature (25 °C). The anti-platelet Ig used was either (I) anti-CD41 (rat monoclonal MWReg30, Pharmingen; 0.2 mg/kg), known to induce immune thrombocytopenia (ITP) [51], or (II) anti-NS1 Ig (rabbit polyclonal, 8.5 mg/kg), which contains anti-platelet Ig fractions and is also ITP-inducible [31]. Anesthesia was induced using isoflurane, a commonly used inhalation anesthetic for mice, 5 min prior to each injection (e.g., vehicle, DENV, and Ig). A mixture of isotype control rat Ig (0.2 mg/kg body weight; Biolegend; vs. MWReg30 anti-CD41 Ig) and Ig from preimmune rabbits (8.5 mg/kg; vs. rabbit anti-NS1 Ig) was used as the control Ig in the dual challenge mouse model.

Twenty-four hours after the injection of anti-platelet Ig (the second hit challenge), which coincides precisely with 48 h following the rEIII treatments (the first hit challenge), we analyzed the platelet counts (using the KX-21N Analyzer, Sysmex; blood samples) [51], the extent of hemorrhage (described in the following section; skin samples), the levels of cytokines TNF-α (ELISA, e-Bioscience, San Diego, CA, USA), IL-1β (ELISA, e-Bioscience), and IL-6 (ELISA, Biolegend), as well as the expression levels of the anticoagulant proteins antithrombin III (chromogenic assay, Sekisui Diagnostica) and protein C (chromogenic assay, Sekisui Diagnostica) using the plasma samples according to previously described methods [35]. For this analysis, whole blood (100 μL) was collected from the mice and mixed with an anticoagulant citrate dextrose solution (38 mM citric acid, 75 mM sodium citrate, 100 mM dextrose) in Eppendorf tubes [51]. Plasma samples were obtained after centrifugation at 2000× *g* for 15 min to remove blood cells and platelets. Additionally, to assess the hemorrhage score, skin samples for imaging the hemorrhagic lesions caused by the dual challenge-induced local Shwartzman reactions were collected from the mice that were euthanized using CO_2_ in compliance with the NIH guidelines (https://oacu.oir.nih.gov/animal-research-advisory-committee-arac-guidelines; accessed on 1 September 2024).

To characterize the endothelial cells in hemorrhagic lesions, we collected mouse skin samples from the areas that had been subcutaneously injected with rEIII and anti-platelet antibodies to induce hemorrhagic lesions following previously established mouse models [25,35]. Skin tissues were formaldehyde-fixed, dehydrated with increasing concentrations of ethanol and xylene, embedded in paraffin wax, and sectioned into 5 μm slices (RM2125RT, Microtome, Leica, Wetzlar, Germany). The sections were mounted on microscope slides and deparaffinized. After deparaffinization, antigen retrieval was performed using Tris-EDTA buffer (10 mM Tris Base, 1 mM EDTA Solution, 0.05% Tween 20, pH 9.0), followed by blocking with 1% BSA/PBS. The sections were incubated overnight at 4 °C with primary antibodies (rat anti-mouse CD31, Biolegend, San Diego, CA, USA; rabbit anti-mouse cleaved caspase-3, Cell Signaling Technology, Danvers, MA, USA). Detection was carried out using Alexa Fluor 594 goat anti-rat IgG (Invitrogen, Life Technologies, Carlsbad, CA, USA) and Alexa Fluor 488 goat anti-rabbit IgG (Invitrogen). Cell nuclei were counterstained with 1 μg/mL of 4′,6-diamidino-2-phenylindole (DAPI; Sigma-Aldrich). The tissue sections were mounted with an aqueous mounting medium (Abcam, Cambridge, MA, USA) and analyzed using a Nikon A1+ confocal microscope (Nikon, Minato, Tokyo, Japan).

Inhibitors and drugs, such as caspase-3 inhibitor Z-DEVD-FMK (R&D Systems, 10 mg/kg), CSB (a glycosaminoglycan that competes rEIII-cell surface glycoprotein binding; Sigma-Aldrich, 0.5 mg/kg), and NAC (a scavenger of ROS; Sigma-Aldrich, 50 mg/kg), were subcutaneously administered 10 min prior to the rEIII injection injections, following described methods [35]. A summary of the experimental settings of the mouse model is available in Figure S3.

### 4.5. Quantification of Hemorrhage Severity Using Digitized Images

In the dual challenge mouse model, rEIII and anti-platelet Ig were sequentially injected subcutaneously at the same skin location on each mouse, with a 24 h interval between injections. Hemorrhage, in this experiment, was a localized response, occurring mainly around the injection sites. Consequently, we collected the skin samples only from around the injection sites. The grading of hemorrhage in the local Shwartzman reaction can typically be assessed using an arbitrary scaling of 0 to 4 [58]. However, in this study, we devised a quantification protocol to achieve a relatively more objective measurement. Images of the hemorrhagic lesions were captured under standardized conditions (ambient light intensity of 200 lux, 20 W Phillips fluorescent lamp; Canon IXUS-860IS camera, with a sample-to-camera distance of 7 cm). Subsequently, red and green signals from the digitized images (RGB mode, 0.75 × 0.6 cm^2^, 600 dpi) were extracted using Photoshop software (version 7.0, Adobe, San Jose, CA, USA) without any adjustments to the brightness or contrast. The red and green intensities in a specific image of the hemorrhagic lesion were further analyzed using Image J software (v1.46, NIH, Bethesda, MA, USA), following methods described previously [35]. A flow chart illustrating the protocol for determining the hemorrhage is available in Supplementary Figure S2.

### 4.6. Statistical Analyses

The experimental data were analyzed using Microsoft Office Excel 2003 and SPSS 17. The statistical significance of the results was assessed through a one-way analysis of variance followed by a post hoc Bonferroni-corrected *t*-test. A significance level of α = 0.05 was adopted as the threshold for determining the statistical significance, with consideration given to the probability of a type 1 error.

## Data Availability

The datasets used and analyzed during the current study are available from the corresponding author upon reasonable request.

## Supplementary Materials

The supporting information can be downloaded at: https://www.mdpi.com/article/10.3390/ijms251910858/s1.
