# Supplementary material for: Dengue Envelope Protein as a Cytotoxic Factor Inducing Hemorrhage and Endothelial Cell Death in Mice
Source: Int J Mol Sci. 2024 Oct 9;25(19):10858. doi: 10.3390/ijms251910858 (PMC11476790; doi:10.3390/ijms251910858)
Supplement: Supplementary file 1 [file ijms-25-10858-s001.zip › ijms-3230632-supplementary.pdf]

Supplemental Data

Dengue Envelope Protein as a Cytotoxic Factor Inducing Hemorrhage and Endothelial Cell Death in Mice

Te-Sheng Lien [1], Der-Shan Sun [1], Wen-Sheng Wu [2,3], and Hsin-Hou Chang [1*]

[1] Department of Molecular Biology and Human Genetics, Tzu-Chi University, Hualien 970, Taiwan.
[2] Division of General Surgery, Department of Surgery, Hualien Tzu Chi Hospital, Buddhist Tzu Chi Medical Foundation, Hualien 970, Taiwan.
[3] Department of Laboratory Medicine and Biotechnology, College of Medicine, Tzu Chi University, Hualien 970, Taiwan.

*Correspondence should be sent to Hsin-Hou Chang.
Department of Molecular Biology and Human Genetics, Tzu-Chi University, Hualien 970, Taiwan, ROC.
Tel: 886-3-8565301 ext 2667. Fax: 886-3-8578386.
E-mail:hhchang@mail.tcu.edu.tw

Figure S1

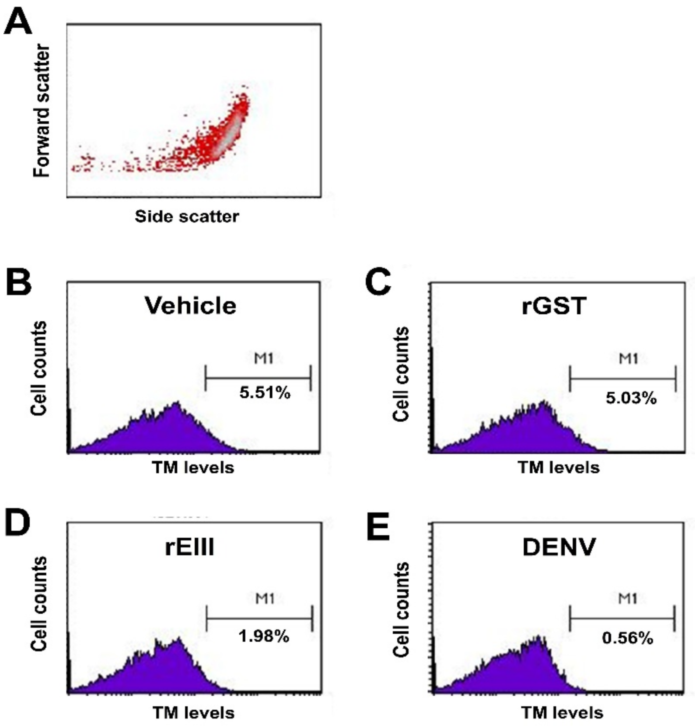

Figure S1. Flow cytometry gating for assessing thrombomodulin expression on endothelial cell surfaces. The graphs display examples of flow cytometry gating used to measure surface thrombomodulin levels on HMEC-1 endothelial cells. (A) Forward scatter and size scatter dot plots. (B-E) Relative cell counts with varying levels of surface thrombomodulin (TM) expression are shown for the vehicle (B), rGST (C), rEIII (D), and DENV (E) treated groups. The TM^{hi} population (B-E, M1 region) was noticeably higher in the vehicle and rGST groups (5.51% and 5.03%, respectively) but decreased following rEIII and DENV treatments (1.98% and 0.56%).

Figure S2

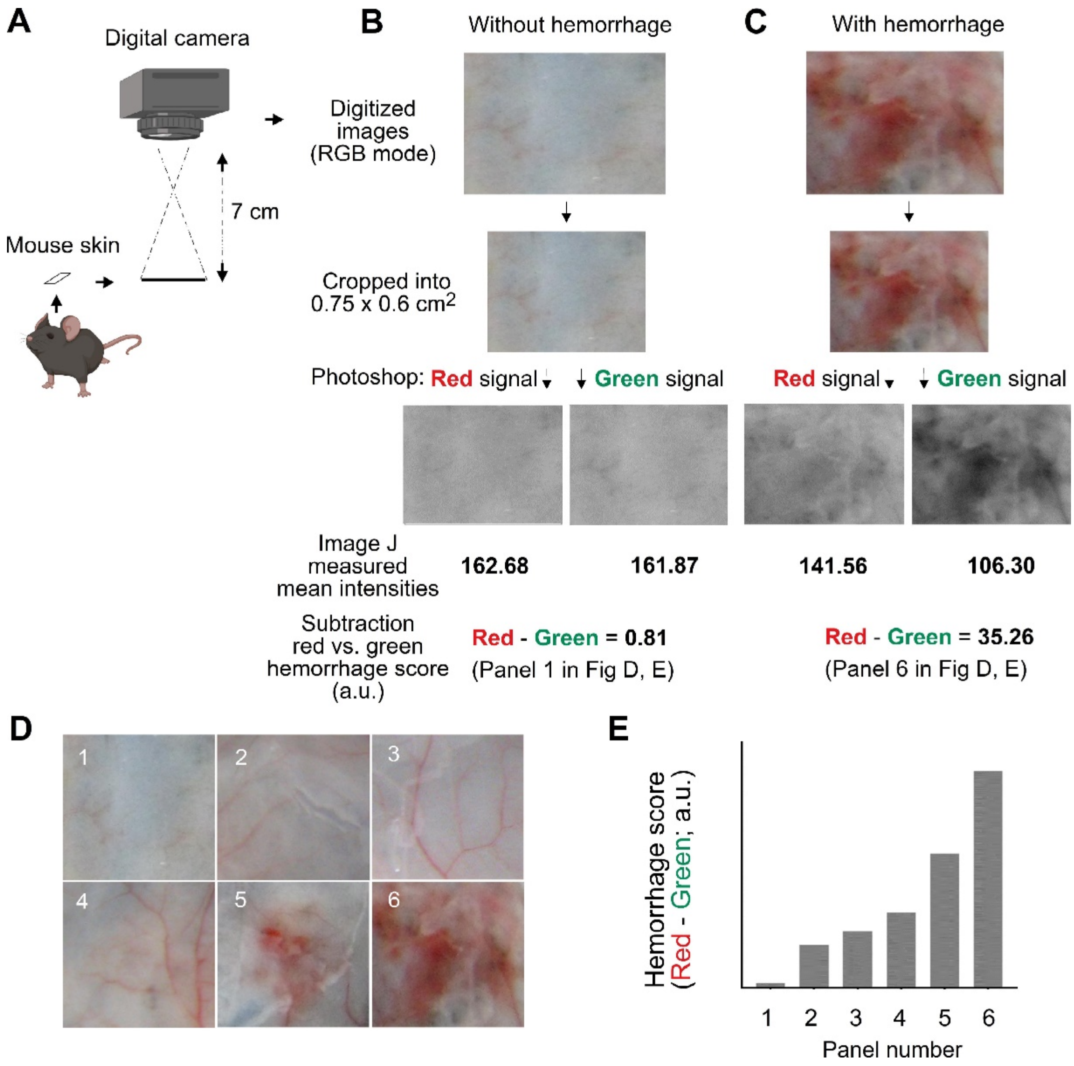

Figure S2. Quantification of combined Effects of dengue envelope protein and autoantibodies induced hemorrhage. We developed an experimental protocol to assess hemorrhage levels in mouse skin (A) and process quantification data (B, C) from images. Photographs were taken in red-green-blue (RGB) color mode under standardized conditions (camera distance: 7 cm, illumination: 200 lux) (A), with no further adjustments to brightness or contrast (B, C). Since red and green are complementary colors, hemorrhage scores (a.u.; relative red intensity and area) were calculated by measuring the mean red channel intensity and subtracting the mean green channel intensity using ImageJ (B, C). Hemorrhage scores for representative images are displayed in panel D (E). The mouse and camera illustrations were created using Biorender.com.

Figure S3

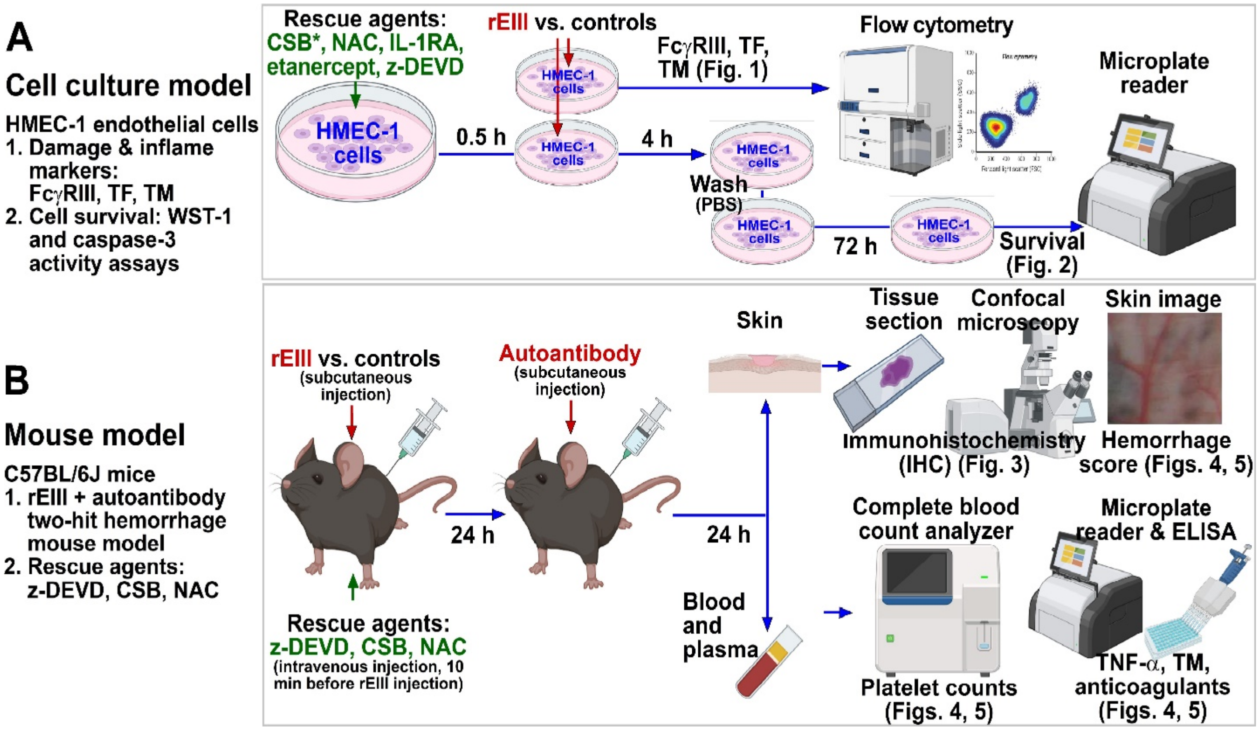

Figure S3. Experiment settings. This illustration summarizes the *in vitro* (A) and *in vivo* (B) models utilized in the study. Cytotoxic agents such as rEIII and autoantibodies are highlighted in red, while cell-protective agents, including z-DEVD (a caspase-3 and apoptosis inhibitor), CSB (an rEIII antagonist), and NAC (an antioxidant), are shown in green. (A) * In HEMC-1 cell survival analyses, a minor protocol difference is noted for CSB: it was dissolved in PBS, co-incubated with rEIII, rGST, and DENV for 0.5 hours in Eppendorf tubes, and then added to the HMEC-1 cells. Other rescuing agents, like NAC, IL-1RA, etanercept, and z-DEVD, were pre-incubated with HMEC-1 cells for 0.5 hours before adding rEIII, rGST, and DENV to the culture. The graph was created using Biorender.com.
